# Heterogeneous Integration of Solid-State Quantum Systems with a Foundry Photonics Platform

**DOI:** 10.1021/acsphotonics.3c00713

**Published:** 2023-08-31

**Authors:** Hao-Cheng Weng, Jorge Monroy-Ruz, Jonathan C. F. Matthews, John G. Rarity, Krishna C. Balram, Joe A. Smith

**Affiliations:** Quantum Engineering Technology Laboratories, H. H. Wills Physics Laboratory and Department of Electrical and Electronic Engineering, University of Bristol, Bristol BS8 1UB, United Kingdom

**Keywords:** nitrogen-vacancy center, silicon nitride, nanodiamonds, heterogeneous integration, solid-state emitters

## Abstract

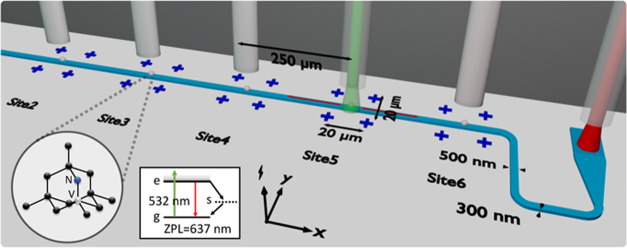

Diamond color centers are promising optically addressable solid-state spins that can be matter-qubits, mediate deterministic interaction between photons, and act as single photon emitters. Useful quantum computers will comprise millions of logical qubits. To become useful in constructing quantum computers, spin-photon interfaces must, therefore, become scalable and be compatible with mass-manufacturable photonics and electronics. Here, we demonstrate the heterogeneous integration of NV centers in nanodiamond with low-fluorescence silicon nitride photonics from a standard 180 nm CMOS foundry process. Nanodiamonds are positioned over predefined sites in a regular array on a waveguide in a single postprocessing step. Using an array of optical fibers, we excite NV centers selectively from an array of six integrated nanodiamond sites and collect the photoluminescence (PL) in each case into waveguide circuitry on-chip. We verify single photon emission by an on-chip Hanbury Brown and Twiss cross-correlation measurement, which is a key characterization experiment otherwise typically performed routinely with discrete optics. Our work opens up a simple and effective route to simultaneously address large arrays of individual optically active spins at scale, without requiring discrete bulk optical setups. This is enabled by the heterogeneous integration of NV center nanodiamonds with CMOS photonics.

## Introduction

Solid-state atom-like systems such as the nitrogen-vacancy (NV) center in diamond show great promise for quantum information.^1,2^ Entanglement state generation^3^ and distillation^4^ have been realized in spin-interfaced quantum networks. Optically addressable spin states operate as quantum registers and memories for quantum computing^5^ and communications.^6^ In addition, NV centers have exceptional characteristics for quantum sensing with high-resolution and high signal-to-noise ratio imaging of electromagnetic fields^7,8^ where introducing spins into photonic waveguides is a prerequisite for emerging applications in quantum sensing.^9,10^ Bringing solid-state systems into foundry photonics would provide the missing component in the integrated quantum photonics toolkit.^11^

Combining atom-like systems into an integrated quantum processor remains a major technological hurdle to date, requiring precise photonic/spin manipulations at scale and the creation of centers at nanoscale resolution.^12^ Miniaturization of photonics and electronics has enabled selective control and manipulation on the same chip.^13,14^ Multiple attempts have been shown to integrate solid-state emitters with in-house-fabricated silicon photonics.^15−18^ A pick-and-place method has been adopted to combine diamond-hosted color centers with integrated photonics.^17,19,20^ However, challenges remain to achieve integration using foundry-manufacturable photonics in a scalable process. In particular, the need to identify^21,22^ and manipulate stochastic emitters^23,24^ requires measurement over sample areas (≈mm^2^) with nanoscale precision and is a highly sensitive and time-intensive technique.

Our approach is to combine NV centers in nanodiamonds (nanoscale inclusions of diamond or NDs) with a standard foundry process. With this heterogeneous approach,^11^ we combine the advantages of diamond as an emitter host with the maturity of a foundry photonics platform. To successfully integrate NV centers with an existing process, it is necessary to select a photonics platform that exhibits moderate refractive index contrast, visibility at the wavelength of interest, and low propagation loss, with mature characteristics and devices. For this, we use IMEC’s BioPIX silicon nitride (SiN) platform with a refractive index of *n* = 1.89 and a propagation loss of 0.9 dB/cm at 638 nm.^25^ The PECVD silicon nitride is deposited at low temperature and is compatible with the necessary control electronics. Of particular importance, this process uses nitrogen-rich SiN, as opposed to standard stoichiometric growth, to reduce the occurrence of defect-based silicon photon emitters. This is critical for its low autofluorescence and compatibility with NV centers.^26^

## Results

**Figure 1:**
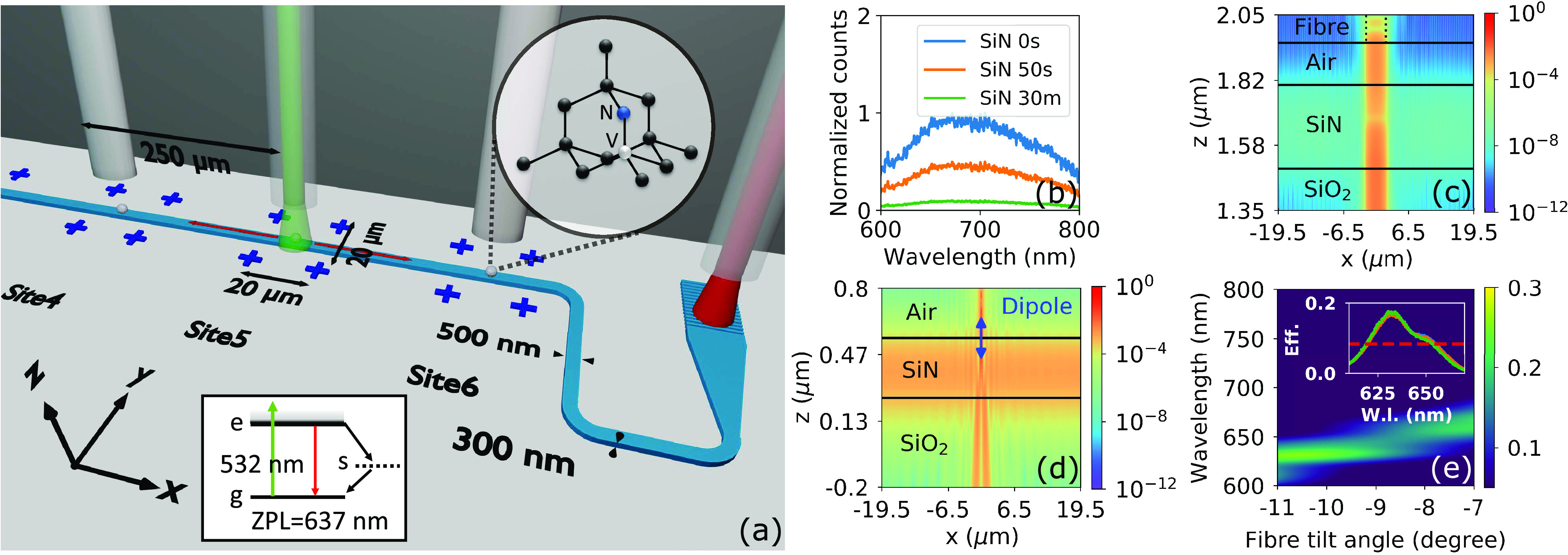
Integration scheme and photonic design. (a) Photonic integration platform of NV centers in NDs. Diagram not to scale. NDs (spheres in white) are positioned over six separate sites on top of a silicon nitride waveguide (in light blue). Each site is defined by four alignment markers (in dark blue). With an array of fibers coupled to the chip (figure upper part), NV centers are excited by a 532 nm (green) laser by selecting one of the middle fibers. The radiated decay of NV is accompanied by PL (in red) emission that is coupled to the waveguide and collected by fibers at the two ends through grating couplers. The NV lattice structure and energy levels are shown in the inset. The coordinate system indicated in the figure will be used throughout the article. (b) Photobleaching SiN fluorescence. The unbleached/0 s, 50 s, and 30 min bleaching results are seen via the SiN fluorescence spectrum scaled according to the bleaching process (Figure S3). Measurement is performed using confocal microscopy. (c) Modeling of spatial filtering of the pump field. A 532 nm laser output from the fiber core (in dashed line) shines directly on the bare SiN waveguide with the SiO_2_ substrate. The power of the electromagnetic field is recorded and also note the highly expanded z-scale. (d) Coupling of an NV center to the waveguide mode. The NV center is modeled by an electric dipole oriented in the z direction at the center of the waveguide. The dipole emission is coupled (from the top) to the SiN waveguide. The power of the electromagnetic field is shown in this figure. (e) Efficiency of a single grating coupler by design. The inset shows the experimental results for three devices on the same chip measured at a 9° fiber tilt.

Here, the NV center integration involves a single postprocessing step with NDs precisely (±200 nm with respect to the waveguide center) and deterministically positioned over millimeter scales, with respect to foundry-defined markers in the SiN (Figure 1a). NV centers in NDs are chosen for the advantage of precise positioning.^24,27,28^ While NDs are primarily used for prototyping here and are in general less favorable emitter hosts than bulk diamond, we note that the spin coherence time for ND-hosted NVs is constantly improving, with *T*_2_ approaching 1 ms,^29,30^ and that broader optical line widths can be overcome with photonic structures in silicon nitride.^17,31^ Moderate refractive index platforms like SiN can support small mode volume cavities that would enable high indistinguishability extraction from even highly dephased emitters.^31^ This integration would be broadly applicable to quantum dots, 2D emitters, or even layers of SiN with intrinsic emitters.^16,32,33^

The manufactured chip consists of four identical devices, each being an air-clad SiN strip waveguide of 500 nm width and 300 nm thickness on top of a SiO_2_ substrate. The waveguides are bent 90° at the ends and tapered to mode-match the grating couplers. Grating couplers are designed to match the PM630 fiber (4 μm mode field diameter). Using nitrogen-rich SiN prevents the pump from creating excess background fluorescence in the waveguide, which greatly diminishes the signal-to-noise ratio in stoichiometric SiN.^26^ In addition, under a strong 532 nm excitation, we observe that the SiN can be bleached permanently, resulting in even lower autofluorescence between 600 and 800 nm. We compare autofluorescence during different stages of this process (Figure 1b). At a 2 mW pump power, a 50 s bleaching reduces 50% of the autofluorescence and a 90% reduction is observed after 30 min.

Six excitation sites are evenly distributed along the waveguide with a 250 μm separation. This enables a scheme to excite NV centers and collect photoluminescence (PL) using a v-groove array of eight fibers (OZ Optics) that couples to the chip. NV centers are excited off-resonantly by a 532 nm pump laser through one of the six excitation fiber channels to emit single photons from lithographically defined locations. Through this all-fiber excitation and collection scheme, we eliminate the need for vibration-sensitive bulk optics. The small cross section between the 4 μm excitation beam and the 500 nm wide waveguide, as well as the orthogonal pump–probe design, results in a strong spatial filtering of the pump. With the bare waveguide, coupling of 532 nm laser to the waveguide and thus fluorescence of SiN in the channel are strongly suppressed by −70 dB as shown in Figure 1c (detailed in Section C of the Supporting Information).

Relaxation from the excited NV center is accompanied by single photon emission in the zero-phonon line (637 nm) and phonon sideband, which is coupled to the evanescent field of the SiN waveguide and guided along its cross section (Figure 1d). The moderate refractive index contrast of SiN-on-silica results in good coupling between the NV center in NDs and the waveguide channel. We find an average of 9% (and a maximum of 28%) coupling efficiency over different positions and orientations of NV centers. Our method improves on integration in low-contrast platforms such as silica^34^ or laser-written diamond waveguides.^35^ PL is guided and collected by fibers at the two ends through grating couplers. The grating coupler is designed around the zero-phonon line with a maximum collection efficiency of 0.25 at 630 nm (Figure 1e). A comparable experimental result shows the maximum efficiency of 0.17 (−7.7 dB) at 630 nm when the fiber is tilted at 9°. The coupling efficiency is repeatable across all of the measured devices. The inset of Figure 1e shows the result of three devices highly overlapped. The grating couplers exhibit a 40 nm bandwidth (fwhm shown by the red dashed line), which helps to filter the 532 nm pump and other background noises.

**Figure 2:**
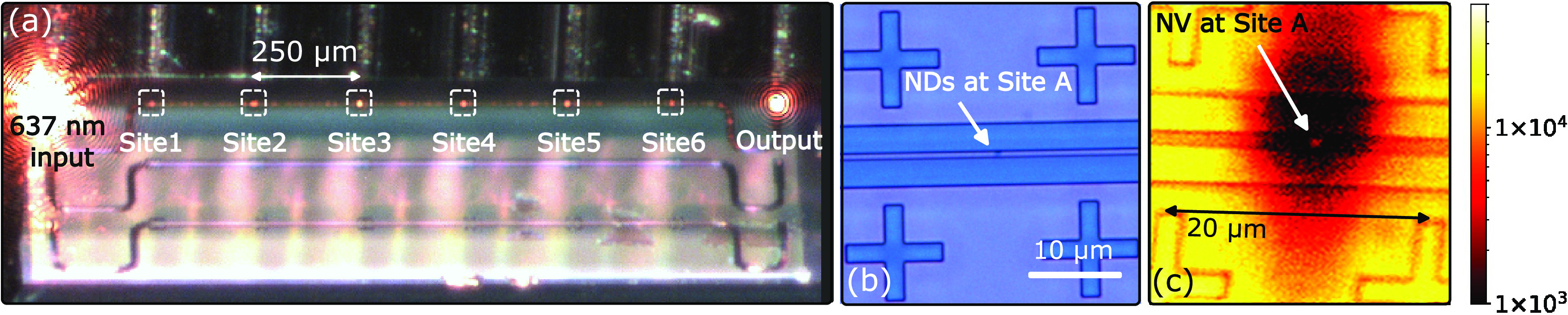
Nanodiamond (ND) positioning on a foundry photonics chip. (a) NDs observed over six sites (in white boxes) of a single device on-chip. A fiber array (figure upper part) is coupled to the photonic chip (figure lower part). NDs on the waveguide are observed by a scattered 637 nm laser. (b) An optical microscope image of Site A where NDs are positioned centrally on top of the waveguide. (c) Confocal microscope PL image of Site A. A contrast between NV PL (3000 counts/s) and the bleached SiN (<400 counts/s) locates the position of NDs on the waveguide. SiN at the site is strongly bleached after exposure to 72 h of a CW 532 nm laser at 6 mW, resulting in the fluorescence level below detector dark counts.

An exemplar device is shown in Figure 2a. The positioned NDs are identified by scattered light when a 637 nm laser is coupled in through the left grating coupler and out through the right grating coupler. The high yield results in NDs at each site (labeled Site1 to Site6). For a site (Site A) studied in the experiment, the positioned NDs are inspected under an optical microscope (Figure 2b) and found well-aligned on top of the waveguide at the center of the site. This is also supported by a fine confocal-scanned image (Figure 2c) with a 1 μm excitation spot size. For a chip of 24 positions over 4 devices, our method presents a 70% yield rate of ND positioning either by identifying through the scattered 637 nm laser or by direct observation under an optical microscope. It should be stressed that the near-unity yield rate of ND positioning is possible.^36^ However, assuming a constant filling rate, our deposition area is chosen to maximize the probability distribution of finding one NV center per site.

To identify that the site-positioned ND contains an NV center that couples to the device, and that this device is successful in both exciting and sufficiently filtering the pump, time-domain photodynamics are measured through the grating output. PL from NV centers exhibits a characteristically long-lived lifetime of tens of nanoseconds during relaxation from the excited state to ground state.^26,37^ We excite the candidate site by a 532 nm pulsed laser through its input port, and the lifetime is measured by coincidence counting between the trigger of the laser and counts detected on a single photon avalanche diode (SPAD) fiber coupled to the grating output. The time-resolved PL is fitted to a biexponential function
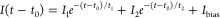
1describing two exponential decays and a constant bias. The decay terms model the NV PL and the background signal of different lifetimes (*t*_1_ and *t*_2_), while the constant bias is a result of detector dark counts. *I*_1_, *I*_2_, and *I*_bias_ are the relative fractions, and the fitting is a function of the relative time *t* – *t*_0_ where *t*_0_ is the pulsed trigger.

**Figure 3:**
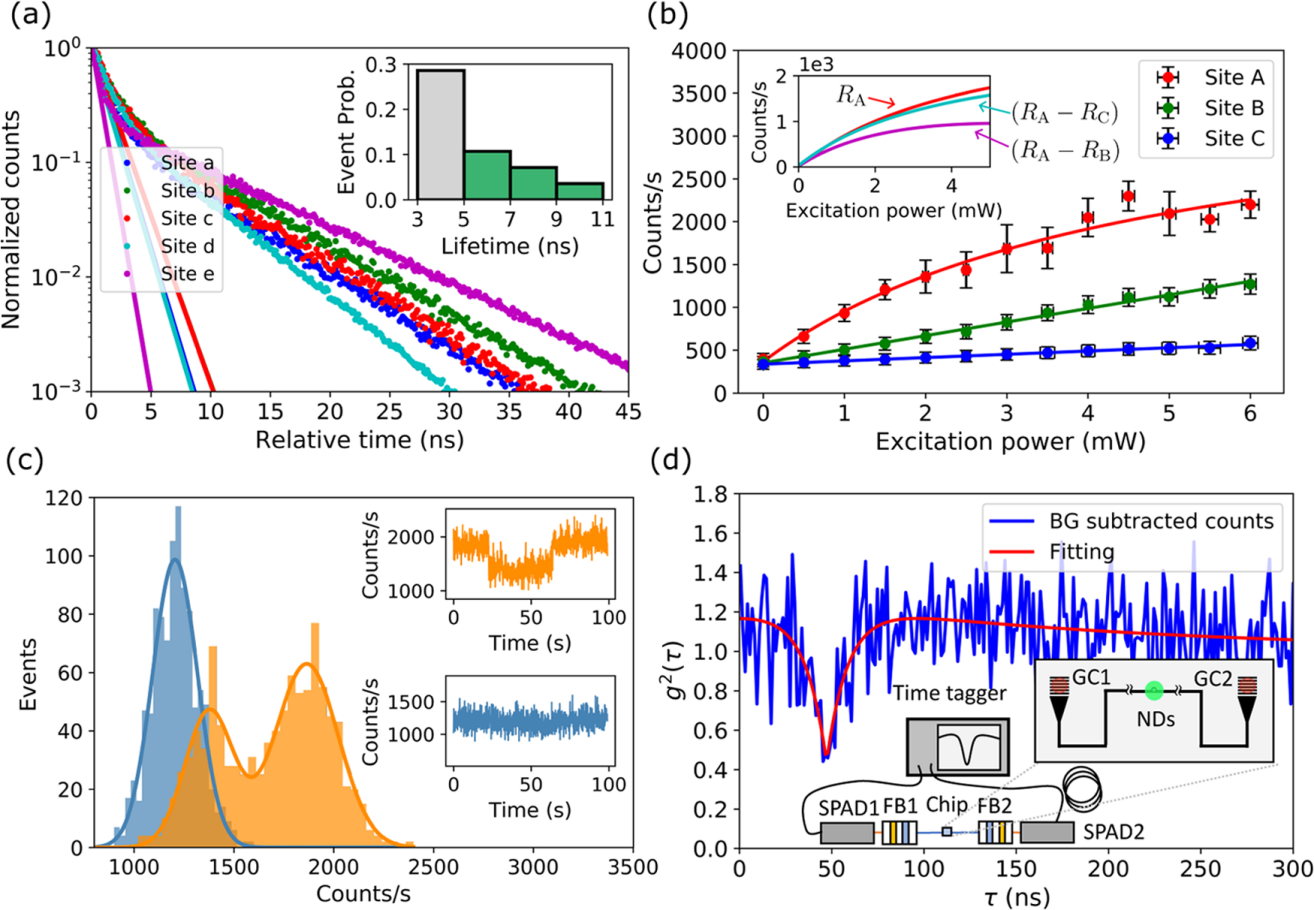
Coupled device results. (a) PL lifetime of five sites containing NV centers. Data points with a detector dark count subtracted. Solid lines are fitted with fast-decay parts. Inset: statistics of lifetimes for all sites containing NDs. (b) Saturated emission. Error bars present the standard deviation of detected counts per second and excitation power. The data points are fitted to eq 2. Inset: saturated emission with background fluorescence subtracted. (c) Blinking in the PL. The count rate per second is recorded for 100 s to show a histogram of count rates and the PL time traces. The blinking (histogram in orange, measured at a 3 mW pump power) and nonblinking (histogram in blue, measured at a 1.5 mW pump power) statistics are compared. (d) HBT experiment. The data are background-subtracted and fitted with eq 3. The inset is the schematic HBT measurement setup. Single photon emission is collected by two grating couplers (GC) separately and fiber-coupled to filter boxes (FB) containing a notch filter and long-pass filter. Single photon counting is time correlated by the time tagger.

For five different sites, the lifetimes (data points) are compared with their fitted fast decaying parts (solid lines), as shown in Figure 3a. The contribution of *I*_bias_ as a result of the detector dark counts has been subtracted. The fitted emission is dominated by a slow decay, which is evidence of PL from NV centers, with lifetime ranging from 5 to 11 ns, as compared with the fast-decay term. This lifetime is evidence of good coupling between the NV center and the waveguide.^26,31^ The fast-decay contribution is a result of background fluorescence, mainly from the excited fiber^38−41^ plus minimal fluorescence contribution from SiN (details in Supporting Information Section C). The background fluorescence falls in the range of 1–5 ns. The statistics of the PL lifetime from NV centers are plotted in Figure 3a, inset, with green bars. PL lifetimes measured shorter than 5 ns are colored in gray as potentially mixed with the background fluorescence term. Conditional on identifying the slow decay PL in a lifetime measurement (44% of the total events fall within green bars in the inset figure), we report on average a 30% (0.44 × 0.70) yield rate of at least one NV center per excitation site.

Another characteristic of NV centers is saturation under strong pumping.^38,42^ We studied saturation by exciting the site with increasing power and detecting PL through one of the grating outputs. In Figure 3b, data is fitted to
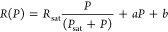
2where *R* describes the emission rate as a function of the laser power *P* saturating at *R*_sat_ with *P*_sat_. Here, *a* models a linear response to the power from the background and *b* models constant dark counts. Three sites on the same device are measured to give reference background fluorescence contributions. NDs are observed optically and by scattered 637 nm laser for both Site A and Site B. A slow PL (of 10 ns lifetime) is measured at Site A but not Site B. The results are compared with a bare waveguide region without NDs (Site C). From the fit, Site A (*R*_A_) clearly saturates, giving further evidence that the signal is dominated by the NV center PL. Counts at Site B (*R*_B_) and Site C (*R*_C_) show linear responses as expected for background fluorescence. The background is much higher with the presence of NDs on top of the waveguide, assumed to be due to additional scattering into the waveguide channel (see Supporting Information Section C). Considering the ND presence, *R*_C_ is regarded as a lower bound to the noise level, and a fair noise estimation at Site A should be *R*_B_. In the Figure 3b inset, the red curve shows *R*_A_ in comparison with the cyan curve (*R*_A_ – *R*_C_) and the magenta curve (*R*_A_ – *R*_B_). The curves are fitted by the saturation model *R*(*P*) = *R*_sat_*P*/(*P*_sat_ + *P*) alone. The net saturated emission is then recovered (combining both grating outputs) with *R*_sat_ = 2600–5500 counts/s and *P*_sat_ = 1.8–3.7 mW. The intervals are lower bound by fitting (*R*_A_ – *R*_B_) and upper bound by (*R*_A_ – *R*_C_). This saturated power corresponds to 1.4 × 10^8^–3.0 × 10^8^ W/cm^2^ power density.

In addition to lifetime and saturation, NV^–^ in small-sized NDs has been shown to exhibit intermittency in the PL (blinking) under a strong excitation of 532 nm. This can be due to the transition between NV^–^ and NV^0^ or other nonradiative decay routes.^43,44^ Under high excitation powers, we measure blinking at Site A through the grating coupler, contributing to further evidence of the NV center-dominated emission. NV centers are excited by a CW 532 nm laser, while the detected count rate per second is recorded for a 100 s interval. In Figure 3c, the count-rate histogram shows double peaks (in orange) when the NV centers are excited with a 3 mW pump power. This is compared with a single peak (in blue) under a 1.5 mW pump. The blinking can also be seen by the time trace of the detected count rates. A sudden decrease in the brightness is followed by recovery after several tens of seconds (Figure 3c, insets).

The defacto method of determining whether emission in a channel arises from a *single* NV center is by observing antibunched statistics through a HBT experiment, where the time-resolved cross-correlation of detection events from two output ports is recorded and normalized to calculate the second-order autocorrelation function *g*^2^(τ). Here, τ is the relative delay between the two detection events after splitting and a dip is expected at *g*^2^(τ = 0). The value at *g*^2^(0) scales for multiemitters following *g*^2^(0) = 1 – 1/*n*, where n is the number of NV centers.^37,42^ As a result, the visibility is diminished when multiple NV centers contribute to the emission. With this device, we perform an on-chip HBT experiment by which a beamsplitter is formed from the NV center coupling to the separate paths of the waveguide (−*x* and *x*). These paths are collected by grating couplers at either end of the device (inset of Figure 3d). One detector is delayed, shifting the dip from τ = 0 to τ = τ_0_ for the resolving of the whole dip. In Figure 3d, the on-chip HBT for Site A is presented. The dip at τ_0_ = 48 ns evidences the quantum statistics of single photon emission. The *g*^2^(τ) is calculated with the background estimated and subtracted. Considering the signal-to-noise ratio σ = *S*/(*S* + *N*), where *S* and *N* are, respectively, the signal and noise, the autocorrelation function under the background noise is related to that without background by *g*_gb_^2^(τ) = 1 – σ^2^ + σ^2^*g*^2^(τ).^45^ From the saturation measurement in Figure 3b, we estimate σ = (*R*_A_ – *R*_B_)/*R*_A_ = 0.42 at a 6 mW pump. We are able to determine that 95% of this noise originates from the fluorescence generated in the excitation fiber (see Supporting Information Section C), with a residual amount contributed by the pump in the waveguide. The result is fitted to a three-level-system model^37,40^
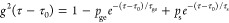
3where the first term describes the transition between the excited state and ground state, while the second term describes with a shelving state. Here, τ_ge_ (τ_s_) and *p*_ge_ (*p*_s_) are the corresponding lifetimes and intensities for each term. From the fit, τ_ge_ is found to be 11 ns, which corresponds well with the 10 ns lifetime measured at Site A. The τ_s_ is found to be 186 ns in good agreement with the literature.^37^ With the background well-estimated and corrected, we measure *g*^2^(0) = 0.48. The same site is measured with a confocal setup showing *g*^2^(0) = 0.46 after background subtraction (Supporting Information Section B), in good agreement. A *g*^2^(0) ≈ 0.5 implies that two NV centers are excited at this site.

## Discussion

**Figure 4:**
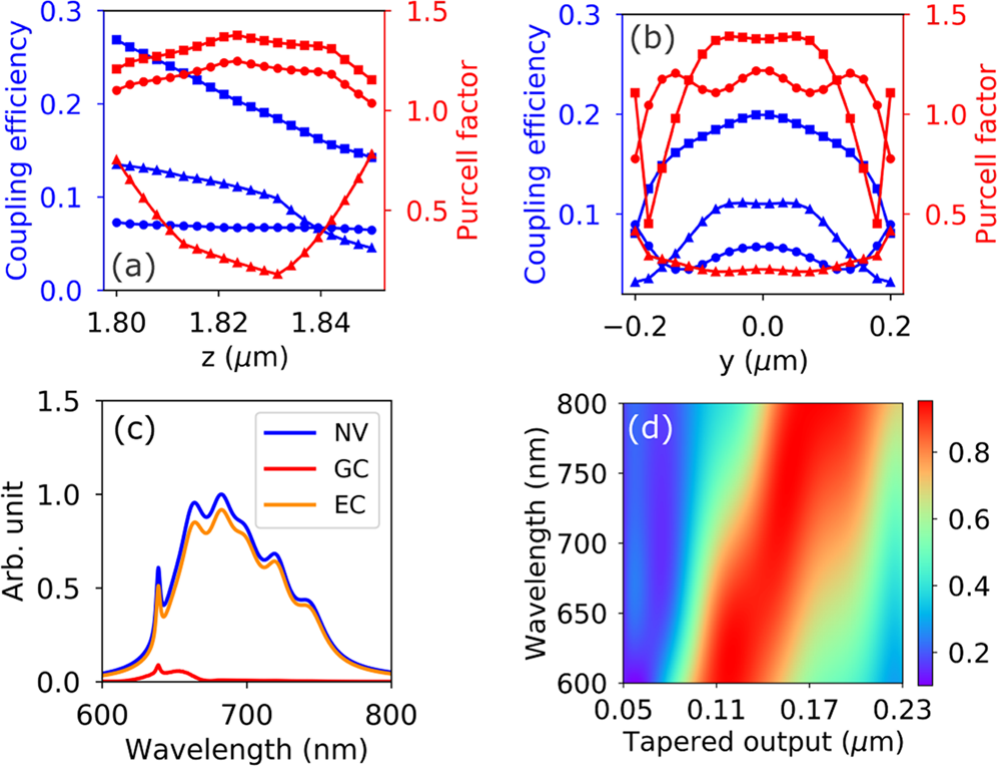
Coupling efficiency and signal-to-noise ratio improvement. (a, b) Coupling efficiency from the NV center to the waveguide and the Purcell factor. The NV center is modeled by an electric dipole source embedded in a 4 μm × 400 nm × 50 nm (*x* × *y* × *z*) region of refractive index 2.4 (diamond) to simulate the nanodiamond deposition area. The coupling is studied when NV is at different depths (in the *z* direction) and displaced from the center (in the *y* direction). The dipole is oriented in the *x*, *y*, and *z* directions for square, round, and triangle markers, respectively. (c) Transmission spectrum of grating couplers (GCs) and edge couplers (ECs) as compared to the NV center PL spectrum. (d) Mode overlap^50^ between the waveguide and fiber mode. The overlapping is calculated for different wavelengths and waveguide-tapered output.

In this first device, the photon flux measured through the waveguide channel is low compared to that of a high-NA confocal microscope, resulting in long integration times for the two-channel HBT measurement. To improve this, the coupled device should be properly modeled. We can describe the detected emission rate *R*_det_ of Site A by
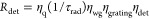
4Here, the internal quantum efficiency of the NV center η_q_ captures decay through nonradiative routes (including the observed blinking). In sub-100 nm NDs, owing to various surface processes, this can range from 0.02 to 0.25^46,47^ so we take a value of . The radiative lifetime τ_rad_ of the NV center is strongly dependent on the surrounding geometry as the local density of optical state (LDOS) can be modified and the emission Purcell enhanced or suppressed.^48^ We can quantify this by the Purcell factor *F* = *P*_act_/*P*_d_ from the actual power radiated normalized by the power radiated in bulk diamond. Simulations of the device find that *F* ranges from 0.2 to 1.4 depending on the position and dipole orientation of the NV center within the deposition region (Figure 4a,b). This variation supports the range measured in Figure 3a with τ_rad_ measured as 10 ns for Site A. Coupling to the waveguide η_wg_ also depends on the orientation of the NV center and its position. Modeled in Figure 4a,b, η_wg_ ranges from 0.03 to 0.27 collecting from both waveguide ends with an averaged value 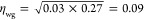
5External efficiencies include the grating couplers designed to capture the zero-phonon line. A reference PL spectrum of the NV center^49^ is shown with its broad phonon sideband in Figure 4c, in comparison with the grating transmission. The area under the curve gives the total efficiency η_grating_ = 0.02 over the spectrum. The final term η_det_ = 0.21 combines the off-chip efficiency including the measured transmission through the filters and the detector efficiency. From this model, *R*_det_ of a single NV center at Site A is estimated as 2650 counts/s. This falls in the range of measured saturated counts for a single NV center of 1300–2750 counts/s, with the assumption that Site A contains two emitters. This is compared with a 40–160 counts/s·mW background fluorescence and 350 counts/s detector dark counts estimated from Figure 3b at each grating coupler.

Considering the contributions to signal loss, the detected emission could be improved by several strategies. One is to pursue edge couplers as opposed to the grating coupler. An advantage of this moderate refractive index platform is that it can be well-matched to the PM630 fiber without lenses or complicated structures. We design a process-compatible edge coupler by tapering the waveguide width from 500 to 150 nm (minimum feature size) and observe a good mode overlap (84%) with the fiber (4 μm diameter) at 637 nm. High efficiency (≥75%) is maintained over a broad bandwidth (Figure 4d). Compared to grating couplers, this leads to a 35-fold improvement in flux across the NV spectrum (Figure 4c). To improve the waveguide mode overlap, we could overcoat the ND in silicon nitride.^26^ We can also underetch the substrate to reduce leakage.^17^ In addition, we could pursue slot waveguides^51^ or nanophotonic cavities to increase Purcell enhancement.^52,53^

Toward quantum photonic applications, we must isolate the remaining background from the measured signal. This contribution is not limited by the low-noise silicon nitride waveguide with fluorescence in the excitation fiber corresponding to 95% of the noise (Supporting Information Section C). By shortening the fiber, adding laser line filters at 532 nm, and switching to photonic crystal fibers,^54^ we can expect strong attenuation of this fluorescence. As shown in Figure 3b, the background observed is proportional to the excitation power. In our scheme, the weak overlap with the 4 μm mode field diameter excitation fiber and the NV center requires significant additional power. In the future, moving to a lens/tapered fiber with a mode field diameter of 1 μm would result in a 16-fold suppression of the background fluorescence. Additionally, we estimate that the fluorescence coupled to the waveguide is tripled in the presence of the positioned NDs, leading to a higher noise level observed in Figure 3b. Considering different diamond geometries, including bulk diamond, could reduce scattering, as well as overgrowing index-matching silicon nitride.^26^

## Conclusions

We have demonstrated the integration of NV centers with foundry photonics where scalable and precise ND positioning is realized in a single postprocessing step. We evidenced NV center coupling through four experimental signatures. Although our work focuses on integrating NV centers, the idea can be generally adopted by other nanocrystal-hosted emitters,^55−58^ opening a route to chip-based integration. The lithographic deposition presented here would be suitable for use in the back end of line foundry processes. We also note that the large cladding opening in the foundry chip is suitable for the heterogeneous integration of pick-and-place diamond or III–V chiplets.^19,59,60^ The all-integrated scheme provides mechanical stability compared to bulk optics or tapered fibers. With edge coupling, an in-plane fiber-terminated device would be suitable for insertion into a cryostat, enabling the generation of narrow linewidth indistinguishable photons. In addition, integrating multiple emitters on the same device demonstrates the idea of routing^61^ and multiplexing^62^ for quantum memories or quantum repeaters. Through foundry compatibility, a scalable platform is envisioned, enriched by the wide range of SiN photonics components and integrated electronics.^63^

## Methods

We use NDs of around 50 nm diameter, milled from high-pressure, high-temperature diamond (Nabond). The concentration of nitrogen atoms shows an averaged probability of 2% to find a single NV center in a single ND.^64^ Integration of NV centers in NDs is achieved in a single lithography deposition step. A poly(methyl methacrylate) (PMMA) mask is first spin-coated on the photonic chip. A 4 μm long and 400 nm wide region (*x* × *y*) is patterned in the middle of alignment markers using electron beam lithography (Raith Voyager). The 400 nm width limits NDs to the center of waveguide, while the long 4 μm region covers the full excitation region of the 532 nm laser. After resist development, NDs in solution are deposited on the chip and left to dry to allow the solvent to evaporate. The PMMA mask is then removed, and NDs remain only over the defined regions on top of the waveguides.

## Supporting Information Available

Main experimental setup, confocal microscopy setup with the supporting HBT measurement result, and noise modeling (to characterize SiN and fiber fluorescence in the system) (PDF)
