# Supplementary material for: Heterogeneous Integration of Solid-State Quantum Systems with a Foundry Photonics Platform
Source: ACS Photonics. 2023 Aug 31;10(9):3302–9. doi: 10.1021/acsphotonics.3c00713 (PMC10515700; doi:10.1021/acsphotonics.3c00713)
Supplement: Supplementary file 1 — ph3c00713_si_001.pdf [file ph3c00713_si_001.pdf]

Supporting Information: Heterogeneous integration of solid state quantum systems with a foundry photonics platform

Hao-Cheng Weng,* Jorge Monroy-Ruz, Jonathan C. F. Matthews, John G. Rarity, Krishna C. Balram, and Joe A. Smith*

Quantum Engineering Technology Labs, H. H. Wills Physics Laboratory and Department of Electrical and Electronic Engineering, University of Bristol, Bristol BS8 1UB, United Kingdom

E-mail: haocheng.weng@bristol.ac.uk; j.smith@bristol.ac.uk

Main experimental setup

For experiments in the main text (Fig.3), NV centres are excited by a pulsed 532 nm laser (PDL 800-B; PicoQUANT) for time resolved PL analysis and a CW 532 nm laser (gem 532; Laser Quantum) in other tests. PL is collected through the two grating couplers in the HBT test or single sided through one grating coupler in other measurements. Coupled PL from the grating coupler passes through an off-chip notch filter (NF01-532U-25; Semrock) and a long pass filter (BLP01-568R-25; Semrockor or FEL-0550; Thorlabs) to block potential pump leakage before detection by single photon avalanche diodes (SPADs)(SPCM-AQRH-12-FC; PerkinElmer). In the HBT test, coincidence counting is measured with a time-correlated single photon counting system (PicoHarp 300; PicoQUANT).

Confocal microscopy setup

A confocal microscope is used to image the PL from NV centres on the chip, to measure the spectrum of SiN fluorescence, and to verify the HBT result. A schematic diagram is shown in Fig.S1 (a). Using a 0.9 NA microscope objective, the excitation beam is highly focused on the sample producing a nearly diffraction-limited spot (< 1 µm diameter) to excite the NV centre on the sample. The NV centre PL is collected through the same lens and separated from the excitation path by the use of a dichroic mirror. By scanning the position of the sample, a map of detected count rate is generated producing the image of the NV centre at Site A (Fig.2 (c) in the main text). Fluorescence of the SiN film is measured when the SiN waveguide is excited and the spectrum of the fluorescence (Fig.1 (b) in the main text) is analyzed using a spectrometer. A HBT measurement is repeated using the confocal setup by splitting the NV emission upon a beam splitter and analyzing the cross correlation of single photon detection. With the confocal setup, we measure a HBT result in Fig.S1 (b) with $g^2(0) = 0.46$ after background corrections.

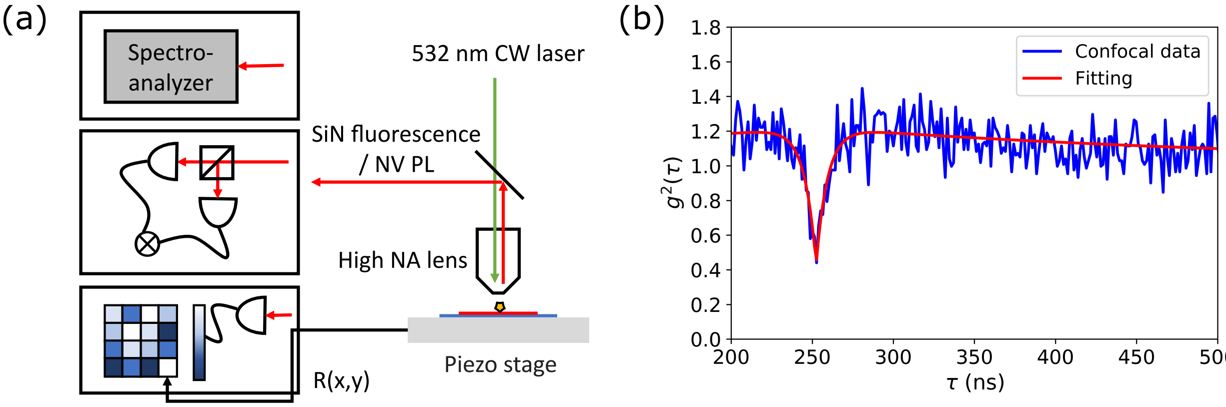

Figure S1: Schematic diagram of the confocal microscopy setup and HBT result. (a) The confocal setup used for the spectral measurement, HBT experiment, and imaging. (b) HBT result as measured by the confocal setup.

Noise modeling

In this section, we model the mechanisms of background noise in our measurements. We show that fluorescence generated in the excitation fibre dominates over the SiN fluorescence in the measured background noise.

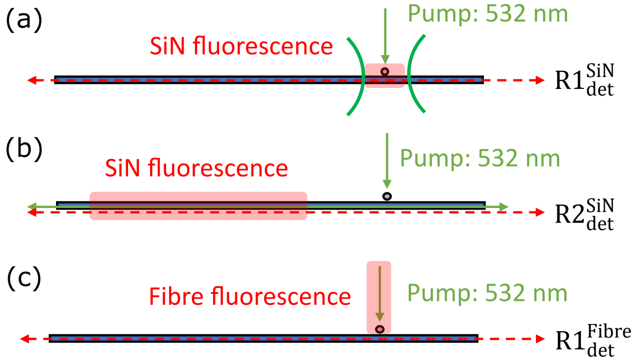

Figure S2: Schematic diagram of noise induced by SiN fluorescence and fibre fluorescence. (a) The first term of the SiN fluorescence $R1^{\mathrm{SiN}}_{\mathrm{det}}$ is where SiN is directly excited by pump (in green) and the SiN fluorescence (in red) is coupled into the waveguide. (b) The second term of the SiN fluorescence $R2^{\mathrm{SiN}}_{\mathrm{det}}$ corresponds to the small fraction of pump is coupled into the waveguide, inducing SiN fluorescence along the waveguide. (c) The fibre fluorescence term $R1^{\mathrm{Fibre}}_{\mathrm{det}}$ refers to the fibre fluorescence generated by the pump propagating in the fibre. This fluorescence is also coupled into the waveguide (alongside the pump) through scattering.

In Fig.3 (b) in the main text, the rate of counts detected at Site B with NDs, compared to the bare waveguide at Site C ($R_{\mathrm{B}} > R_{\mathrm{C}}$), implies that an increased amount of pump or potential fibre fluorescence is coupled into the waveguide when scattered by the NDs. This is compared to -70 dB coupling in the case of a bare waveguide (Fig.1 (c) in the main text).

In our model, we also estimate the scattering-caused enhancement of coupling. Our estimated noise level agrees with experimental results observed from Fig.3 (b) in the main text.

The noise contribution from SiN fluorescence is modeled by two terms $R1^{\mathrm{SiN}}_{\mathrm{det}}$ and $R2^{\mathrm{SiN}}_{\mathrm{det}}$. The first term (as shown in Fig.S2 (a)) considers the region of SiN waveguide directly excited by the 532 nm pump right at the fibre output. SiN fluorescence is generated in this region and coupled to the waveguide. For the second term (Fig.S2 (b)), a small amount of 532 nm pump is coupled to the waveguide, especially when scattered by NDs. The coupled

pump induces SiN fluorescence while propagating along the waveguide. For both terms, the detected count rate for a single grating coupler output (at 6 mW pump) is calculated as:

$$\begin{aligned} R1^{\mathrm{SiN}}_{\mathrm{det}} &= R^{\mathrm{SiN}}_1 \, \eta^{\mathrm{SiN}}_{\mathrm{wg}} \, \eta^{\mathrm{SiN}}_{\mathrm{grating}} \, \eta_{\mathrm{det}} \\ &= 2.7 \times 10^{-13} \alpha^{\mathrm{SiN}}_1 \text{ Hz}, \end{aligned} \tag{S1}$$

$$\begin{aligned} R2^{\mathrm{SiN}}_{\mathrm{det}} &= R^{\mathrm{SiN}}_2 \eta^{\mathrm{SiN}}_{\mathrm{wg}} \, \eta^{\mathrm{SiN}}_{\mathrm{grating}} \, \eta_{\mathrm{det}} \\ &= 2.9 \times 10^{-16} \gamma \alpha^{\mathrm{SiN}}_2 \text{ Hz}. \end{aligned} \tag{S2}$$

where, common to both equations, $R^{\mathrm{SiN}}_{1(2)}$ is the fluorescence count rate (in unit of Hz) generated by excited SiN. $\eta^{\mathrm{SiN}}_{\mathrm{wg}}$ is the coupling efficiency of SiN fluorescence to the waveguide, split into the x and $-x$ direction and is estimated by simulating dipole emission embedded in the SiN waveguide. $\eta^{\mathrm{SiN}}_{\mathrm{grating}}$ is the collection efficiency of SiN fluorescence by the grating couplers. The broad spectrum of SiN fluorescence (Fig.1 (b) in the main text) results in a grating coupler efficiency $\eta^{\mathrm{SiN}}_{\mathrm{grating}} = 0.03$. The off-chip detection efficiency remains as $\eta_{\mathrm{det}} = 0.21$.

In Eq.S1 and Eq.S2, the fluorescence count rate can be found as:

$$R^{\mathrm{SiN}}_{1(2)} = \alpha^{\mathrm{SiN}}_{1(2)} E_{\mathrm{p1(p2)}} L A, \tag{S3}$$

where $\alpha^{\mathrm{SiN}}_{1(2)}$ (in unit of Hz/W·m) is the SiN material response under 532 nm excitation per unit length(depth), $E_{\mathrm{p1(p2)}}$ is the power density of excitation beam(in unit of W/m^2), L is the penetration depth of pump (in meter) and A is the interaction area (in m^2).

As SiN is bleached at the excitation site, this implies $\alpha^{\mathrm{SiN}}_1 \ll \alpha^{\mathrm{SiN}}_2$. In Fig.S3 (a) we measure these bleaching dynamics using a confocal setup when SiN waveguide is excited by 2 mW pump. A long-term exposure to high-power pump results in permanent and strong bleaching of SiN. The value of $\alpha^{\mathrm{SiN}}_{1(2)}$ is back calculated from confocal measurement when the SiN waveguide is directly excited. The detected count rate from a bleached region is

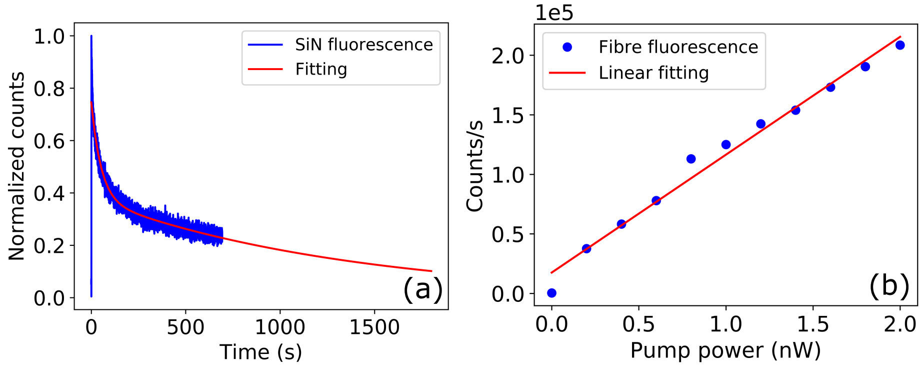

Figure S3: Background fluorescence quantification. (a) Bleaching dynamics of SiN fluorescence. The blue curve is measured experimentally and well fitted (in red curve) to a double exponential decay function. (b) Fluorescence from a 5 meter PM630 fibre proportional to the pump power.

compared with non-bleached one as:

$$R_{\text{bleached}} = \eta_{\text{conf}}\ \alpha_1^{\text{SiN}} E_{\text{p}} L A, \tag{S4}$$

$$R_{\text{non-bleached}} = \eta_{\text{conf}}\ \alpha_2^{\text{SiN}} E_{\text{p}} L A. \tag{S5}$$

We record $R_{\text{bleached}} = 200$ Hz (from Fig.2 (c) in the main text, subtracting 200 Hz detector dark counts) and $R_{\text{non-bleached}} = 8000$ Hz at 0.6 mW pump. With the setup efficiency $\eta_{\text{conf}} = 0.01$, we estimate the SiN response $\alpha_1^{\text{SiN}} = 1.8 \times 10^{14}$ and $\alpha_2^{\text{SiN}} = 7 \times 10^{15}$.

For R_2^{SiN}, we have $E_{\text{p2}} = 4 \times 10^{10} \gamma \eta_{\text{sc}}$ with the pump-to-waveguide coupling efficiency $\eta_{\text{sc}} = 10^{-7}/2$ and the scattering-caused enhancement factor γ. The value of γ will vary dependent on the exact make-up of nanodiamonds at each site. $R1_{\text{det}}^{\text{SiN}}$ is thus estimated to be 8 Hz and $R2_{\text{det}}^{\text{SiN}} = 0.3\gamma$ Hz, at 1 mW pump.

As shown in Fig.S2 (c), we expect a strong component of the fluorescence, and hence the measured noise, has been generated externally as the 532 nm laser propagates in the fibre which is then coupled into the SiN waveguide. The detected count rate of fibre fluorescence

$R1^{\mathrm{Fibre}}_{\mathrm{det}}$ is described by

$$R1^{\mathrm{Fibre}}_{\mathrm{det}} = R^{\mathrm{Fibre}}\ \eta^{\mathrm{Fibre}}_{\mathrm{wg}}\ \eta^{\mathrm{Fibre}}_{\mathrm{grating}}\ \eta_{\mathrm{det}} \\ = 5.2 \times 10^{-10} \gamma R^{\mathrm{Fibre}} \eta_{\mathrm{det}}\ \mathrm{Hz}. \tag{S6}$$

Here, R^{Fibre} is the rate of fibre fluorescence generated. $\eta^{\mathrm{Fibre}}_{\mathrm{wg}} = \eta_{\mathrm{sc}}\gamma$ is the coupling of fibre fluorescence to waveguide, with the same waveguide coupling efficiency $\eta_{\mathrm{sc}} = 10^{-7}/2$ and scattering enhancement factor γ as Eq.S2. $\eta^{\mathrm{Fibre}}_{\mathrm{col}} = 0.01$ is calculated from the spectrum of fibre fluorescence and $\eta_{\mathrm{det}} = 0.21$ as before.

Experimentally, in Fig.S3 (b), we measure the fluorescence of the fibre directly by SPADs at different pump powers using the same pump filter as the main experiment. With the fitted gradient, we extract $R^{\mathrm{Fibre}}\eta_{\mathrm{det}} = 6 \times 10^{11}$ Hz for a 6 mW pump power. From this, $R1^{\mathrm{Fibre}}_{\mathrm{det}} = 52\gamma$ Hz is back calculated for a 1 mW pump.

In conclusion, one can compare the three terms− $R1^{\mathrm{SiN}}_{\mathrm{det}}$ =8 Hz/mW, $R2^{\mathrm{SiN}}_{\mathrm{det}} = 0.3\gamma$ Hz/mW, and $R1^{\mathrm{Fibre}}_{\mathrm{det}} = 52\gamma$ Hz/mW. As the enhancement factor γ caused by ND scattering is defined to be greater than one, this implies the fluorescence of fibre dominates in the noise contribution. We estimate the scattering enhancement factor $\gamma = 3$, corresponding to a noise level of 60 Hz/mW without NDs ($\gamma = 1$) and 165 Hz/mW with NDs ($\gamma = 3$). Contribution of each term is shown in Table S1 (with $\gamma = 3$). One should note that the fibre fluorescence contributes to 95% (156/(156+8+0.9)) of the noise level. This generally agrees with the experimental observation of 40 Hz/mW (without NDs) and and 160 Hz/mW (with NDs).

Table S1: The three terms of noise background $R1^{\text{SiN}}_{\text{det}}$, $R2^{\text{SiN}}_{\text{det}}$, and $R1^{\text{Fibre}}_{\text{det}}$ in comparison (considering $\gamma = 3$).

	$R1^{\text{SiN}}_{\text{det}}$	$R2^{\text{SiN}}_{\text{det}}$	$R1^{\text{Fibre}}_{\text{det}}$
$R^{\text{SiN}}_{1(2)}$ @ 6 mW	5×10^4 Hz	6.3×10^3 Hz	-
R^{Fibre} @ 6 mW	-	-	2.9×10^{12} Hz
$\eta^{\text{SiN}}_{\text{wg}}$	0.15	0.15	-
$\eta^{\text{Fibre}}_{\text{wg}}$	-	-	1.5×10^{-7}
$\eta^{\text{SiN}}_{\text{grating}}$	0.03	0.03	-
$\eta^{\text{Fibre}}_{\text{grating}}$	-	-	0.01
η_{det}	0.21	0.21	0.21
R_{det} @ 1mW	8 Hz	0.9 Hz	156 Hz
